# Dr. Huggan, on Mercury in Hydrophobia

**Published:** 1805-12

**Authors:** A. Huggan

**Affiliations:** Wallingford


### *To the Editors of the Medical and Physical Journal.*

GENTLEMEN,

I TAKE the liberty of reviving the discussion of Hydrophobia, which I am sorry was so soon dropped, trusting that the importance of the subject will be a sufficient apology for my intrusion.

I shall begin with remarking on the benefit which Dr. Bardsley seems to expect from the addition of arsenic to stimulants in the treatment of this disease, (Medical and Physical Journal, No. 72, p. 163.) that, I cannot reconcile a dose of it that is active (one that produces a sensible effect on the constitution,) with its being safe; nor, that the slow and imperceptible action of a dose of arsenic that is merely *safe,* can either promote or increase the stimulant property of other articles, particularly in a disease which so speedily extinguishes life.

What encouragement Mr. Hope could give to this practice, by his communication of the Colthorpe receipt, &c. has already been shown by an abler pen than mine; it is needless for me, therefore, to notice it further, than to add, that arsenic when found with tin or pewter is in a metallic state, and as inert, with respect to the constitution, as lead, mercury, or any other metal; therefore, whatever virtue pewter may be supposed to possess beyond its mechanical action upon the stomach, no part of it need be ascribed to the arsenic, with which it may be accidentally combined.

But I now proceed to the more immediate object of this letter, which is, to propose *Mercury* as a preventive of hydrophobia, in cases where the destruction of the bitten part cannot be done safely, or, what is tantamount, where it has been neglected.

Mercury, as employed with this view, is certainly founded on analogy; but if my authority is to be credited, it is also supported by experience.

It is easy to trace the analogy between hydrophobia and syphilis, as both affect the constitution through the medium of an external and specific inflammation; and "both may be prevented, by preventing this external and specific inflammation;" or by *immediately* destroying it: and I may add, that, the interval between the external or exciting cause and the constitutional disease, in both is nearly the same. From all this, may we not reasonably conclude, conclude, that hydrophobia is as likely to be prevented by a course of mercury as syphilis ?

The argument is further strengthened from the prophylactic power of mercury in another disease, to which, though it is not of animal origin, hydrophobia is still more closely allied, I mean tetanus, as it appears in the West Indies.

Both are sometimes spontaneous, but oftener they arise from an obvious and external cause ; both are diseases of nervous irritation ; both may be prevented by destroying the external *medium* through which they affect the constitution ; both are distinguished by a peculiar spasmodic stricture, or convulsion ;—in hydrophobia, about the fauces on the sight of liquids ; in tetanus, in the course of the diaphragm on the slightest motion or effort of the patient ; both are terminated in nearly the same time, and for the most part in the same way ; tetanus is generally, hydrophobia is always, fatal.

The most obvious causes of tetanus are fractures, amputation, (except at the joints) and punctures in the soles of the feet, from fish bones, rusty nails, and the like ; in such instances of puncture, the disease may in general be prevented, by laying the punctured part fully open, as recommended by Dr. Mosely, (in his Treatise on Tropical Diseases) and by exciting suppuration as quickly as possible, thus prevent the *specific* irritation, which constitutes the first link in the morbid catenation : no doubt, excision, or the potential cautery, would answer the purpose as effectually.

That excellent practical physician Dr. Clarke, of Dominica, having observed that when tetanus followed amputation, it generally came on about the seventh or eighth day after the operation, was induced to try if it could not be prevented by interposing a course of *Mercury*, so as to excite a slight ptyalism within that period, and in which he has generally succeeded.

Though the interval, when it is occasioned by a puncture, as mentioned above, has not been so precisely ascertained ; yet as I have some reason for supposing that it is nearly the same, or a little longer, it may be presumed that mercury, judiciously administered as a preventive, would produce the same beneficial effect.

Tetanus has sometimes been cured by mercury ; but it must also be acknowledged, that it has proved fatal, even after the specific irritation of meacury, in the system had been excited.

I need

I need not pursue this part of the subject any further, but go on to show, on what practical authority mercury can be recommended as a preventive in hydrophobia; and to this point shall mention first, the cases of Lieutenants A. and B. of the 36th regiment of foot, when it was stationed in India under Lord Cornwallis. Both these gentlemen were bitten, by a dog belonging to Lieutenant A. that was mad, though not suspected to be so at the time; however, that this might be ascertained, he was confined, and died two or three days afterwards, with every appearance of the disease. Mr. A. now became apprehensive of the consequence, and applied to Mr. H. the regimental surgeon, a very well informed man, and always attentive to every part of his duty. Mr. H. advised him, as it was now too late, *in his opinion*, to expect any benefit from the destruction of the bitten part, to undergo a course of mercury, as the only chance of safety that he now had, with which he complied, and persevered in it upwards of two months.

On the other hand, Lieutenant B. not only obstinately refused to use the same *preventive* means, but affected to laugh at the fears of his friend, and ridicule the *discipline* which he was about to undergo, as quite *unnecessary:* so true it is,

*Quem Deus vult perdere, prius dementat.*

Sometime after this, one day, when this gentleman went a hunting with a party of his brother officers, on coming to a rivulet or other piece of water, which they easily crossed, he found himself *unaccountably* unable to follow them, therefore turned back. Not willing, however, to be thought unwell, he went to dinner in the mess-room, but found his appetite quite gone; and on being asked to drink a glass of wine, he tried to raise it to his mouth, but found his jaws convulsively shut against it, was therefore obliged to set it down, saying, in a peevish tone of voice, that he could not take it. He sat in a sullen mood all the afternoon, now and then involuntarily exclaiming, that he felt himself much indisposed, but did not seem at all apprehensive of the nature of his complaint. As he was going out to the evening parade with the other officers, there was a slight shower of rain, when he was instantly seized with a fit of delirium, or phrenzy, and pulling his coat over his head, ran to his barrack-room, shrieking horribly all the way.

Mr. A. who had the fortune to escape the same fate, was accidentally killed about a year afterwards; and much much about the same time the surgeon was surprised by a party of Tippoo Saib's banditti, who put him to death.

I had these cases from a very respectable and intelligent gentleman, a field officer in the same regiment, and who was present when these circumstances happened.

Professor Harwood, as I have understood, kept a person who had been bitten by a mad dog, under a course of mercury for three months, for the purpose of preventing hydrophobia, and succeeded.

Dr. Reid,* of Ramsgate, has giving several very striking instances of the prophylactic power of mercury, in several people who had been bitten by a mad dog, and of dogs also, that were saved by this means, whilst others, bitten by the same dog, to which it was not given, perished.

But to conclude, whatever may be expected from mercury, with the view here proposed, I readily acknowledge that I should not even propose it, (at least not yet,) unless I was convinced that the destruction of the bitten part was impracticable, or the patient would not submit to it.

Though I am sensible of the danger of speculating upon the cure of hydrophobia, yet I must here take the liberty of adding, that, if ever a case of hydrophobia should come under my care, I think, that along with electricity and other stimulants, I should be induced to try the effect of mercury, in every form in which it can be administered, to excite, if possible, its specific effect, as, in my opinion, the most likely means of saving the patient's life.

I am, &c.

*Wallingford, Nov.* 14, 1805. A. HUGGAN, M. D.

